# Direct, Continuous Monitoring of Air Pollution by Transgenic Sensor Mice Responsive to Halogenated and Polycyclic Aromatic Hydrocarbons

**DOI:** 10.1289/ehp.10722

**Published:** 2007-12-20

**Authors:** Ayumi Kasai, Nobuhiko Hiramatsu, Kunihiro Hayakawa, Jian Yao, Masanori Kitamura

**Affiliations:** Department of Molecular Signaling, Interdisciplinary Graduate School of Medicine and Engineering, University of Yamanashi, Yamanashi, Japan

**Keywords:** aromatic hydrocarbon, aryl hydrocarbon receptor (AhR), cigarette smoke, dioxin-responsive element (DRE), secreted alkaline phosphatase (SEAP), transgenic mouse

## Abstract

**Background:**

The aryl hydrocarbon receptor (AhR, also called the dioxin receptor) plays crucial roles in toxicologic responses of animals to environmental pollutants, especially to halogenated and polycyclic aromatic hydrocarbons. To achieve direct, continuous risk assessment of air pollution using biological systems, we generated transgenic sensor mice that produce secreted alkaline phosphatase (SEAP) under the control of AhR.

**Methods:**

To characterize responses of the mice to AhR agonists, sensor mice were orally administered 2,3,7,8-tetrachlorodibenzo-*p*-dioxin (TCDD), 3-methylcholanthrene (3MC), benzo[*a*]pyrene (B[*a*]P), or β-naphthoflavone (BNF), and serum levels of SEAP were evaluated. To monitor air pollution caused by cigarette smoke, we placed the mice each day in an experimental smoking room, and evaluated activity of serum SEAP for up to 4 days. Activation of AhR in individual organs was also examined by reverse transcription–polymerase chain reaction (RT-PCR) analysis of *SEAP*.

**Results:**

In response to oral exposure to TCDD, sensor mice exhibited dramatic and sustained activation of AhR. The mice also responded sensitively to 3MC, B[*a*]P, and BNF. Activation of AhR was dose dependent, and the liver was identified as the main responding organ. After exposure to the smoking environment, sensor mice consistently exhibited transient, reversible activation of AhR. RT-PCR analysis of *SEAP* revealed that activation of AhR occurred predominantly in the lung.

**Conclusion:**

We are the first laboratory to demonstrate successfully direct, comprehensive monitoring of air pollution using genetically engineered mammals. The established system would be useful for real risk assessment of halogenated and polycyclic aromatic hydrocarbons in the air, especially in smoking environments.

Halogenated and polycyclic aromatic hydrocarbons, two of the most extensively studied groups of ubiquitous environmental pollutants, cause a broad spectrum of toxicity in mammals, including carcinogenesis, teratogenesis, and immune dysfunction (Poland and Knutson 1982). Most of these toxic effects are mediated by the cytosolic receptor—the aryl hydrocarbon receptor (AhR). Once these xenobiotics are recognized by AhR, the AhR–ligand complexes translocate into the nucleus and form heterodimers with its partner coactivator, AhR nuclear translocator (Arnt). The AhR–Arnt heterodimers then bind to their recognition sequence, the dioxin-responsive element (DRE) (Mimura and Fujii-Kuriyama 2003). This molecular event results in transcriptional induction of downstream genes such as cytochrome P4501A1 (*CYP1A1*) that may generate mutagenic metabolites and reactive oxygen species (Nebert et al. 2000), leading to cellular activation, dysfunction, and/or programmed death. Previous reports showed that transgenic mice with constitutively active AhR exhibited malignant tumors, immune abnormalities, and spontaneous inflammation (Andersson et al. 2002; Nohara et al. 2005; Tauchi et al. 2005). Furthermore, other investigators have also shown that AhR-deficient mice are resistant to aromatic hydrocarbon-induced carcinogenesis and teratogenesis (Mimura et al. 1997; Shimizu et al. 2000), as reviewed by our group (Kitamura and Kasai 2007). Monitoring of environmental pollution by halogenated and polycyclic aromatic hydrocarbons is, therefore, important for promotion and maintenance of human health.

Contamination of food, water, soil, and air by aromatic hydrocarbons is monitored using physicochemical methods such as gas chromatography–mass spectrometry to measure the levels of individual pollutants. However, this conventional approach has obvious limitations in estimating the real risk of environmental pollution in humans. For example, this approach does not consider relative bioavailability and metabolism of these toxicants in mammals. It is also unable to consider synergism and antagonism among various compounds, although environmental samples contain numerous toxic and nontoxic substances. Furthermore, in daily life, we are continuously exposed to a wide range of pollutants through respiratory, ingestive, and transdermal routes. However, the current monitoring method does not allow for direct assessment of accumulative and integrative influences of xenobiotics on human health. To overcome these problems, we generated transgenic sensor mice that produce secreted alkaline phosphatase (SEAP) under the control of AhR (Kasai et al. 2006a). Using these mice, we demonstrate for the first time the feasibility of direct, comprehensive, and real-time monitoring of the levels of dioxin-like substances in air via simple blood sampling.

## Materials and Methods

### Reagents

2,3,7,8-Tetrachlorodibenzo-*p*-dioxin (TCDD), benzo[*a*]pyrene (B[*a*]P), and β-naphthoflavone (βNF) were purchased from Wako Pure Chemical Industries (Osaka, Japan). Corn oil and 3-methylcholanthrene (3MC) were obtained from Sigma-Aldrich Japan (Tokyo, Japan).

### Generation of DRE-based sensing via SEAP (DRESSA) mice

The *Mlu*I*-Sal* I fragment of pDRE-SEAP (Kasai et al. 2004) was microinjected into the pronuclei of fertilized oocytes of C57BL/6J mice. Transgenic pups were screened by polymerase chain reaction (PCR) using the following primers purchased from Sigma-Aldrich Japan: forward primer 5′-CAGGACATCGCTACGCAGCTCATCT-3′; reverse primer 5′-GTAAGCC CTGCTTTCATGATGACCA-3′.

Two transgenic lines, DRESSA24 and DRESSA25, were established by our laboratory. Males responded more sensitively to TCDD than females. Adult male DRESSA25 mice (heterozygous) (Kasai et al. 2006a) were generally used for studies

### Administration of AhR agonists to mice

Using feeding needles, we administered 0.1–5 μg/kg body weight (bw) TCDD in 0.25–0.5 mL corn oil orally to 9- to 10-week-old mice or 10 mg/kg bw in 0.25–0.3 mL corn oil of other AhR agonists, including 3MC, B[*a*]P, and βNF. We obtained blood samples (~ 20 μL) from tail veins to evaluate levels of serum SEAP, as described previously (Hiramatsu et al. 2005, 2006).

### Exposure of mice to polluted air

We placed mice in an experimental smoking room (60 × 40 × 35 cm) for 3 hr. During the experiment, the room was ventilated continuously with fresh air, using a mini-pump (1 L/min; GEX, Inc., Osaka, Japan). Mainstream smoke (250 mL) prepared from cigarettes containing 14 mg tar (Seven Stars; Japan Tobacco, Inc., Tokyo, Japan) was injected into the room every 30 min for a total of 6 times/3 hr during the exposure period. Kinetics of smoke density in the room was measured by Digital Aerosol Monitor Model 3411 (Kanomax, Osaka, Japan). After exposure to polluted air for 3 hr, mice were maintained for the following 21 hr outside the smoking room. This procedure was repeated every 24 hr for up to 4 days. Blood sampling was performed before and 0, 3, 6, 9, and 21 hr after the last exposure to polluted air. For the control group, DRESSA mice were similarly kept for 3 hr in the smoking room without exposure to smoke. To analyze expression of *SEAP* and *Cyp1a1* in various organs, we exposed mice to polluted air for 3 hr, as described above. One hour after the last exposure, mice were sacrificed, and organs were subjected to extraction of RNA and reverse transcription (RT)-PCR as described in a later section. Animal experiments were performed according to regulations and guidelines at the University of Yamanashi. Animals were treated humanely and with regard for alleviation of suffering.

### Chemiluminescent assay

We evaluated the activity of SEAP in serum using chemiluminescence (Great EscAPe SEAP detection kit; BD Bioscience, Palo Alto, CA, USA), as described previously (Kasai et al. 2005).

### Activity of SEAP in individual organs

Wild-type mice and DRESSA24 were perfused with phosphate-buffered saline to remove blood completely; protein extracts from brain, lung, heart, liver, spleen, and kidney were subjected to the chemiluminescent assay. We calculated activity of SEAP per 1 mg total protein and evaluated its relative levels versus those in wild-type mice.

### RT-PCR

We extracted total RNA using TRIzol (Invitrogen, Carlsbad, CA, USA), an EZ1 RNA kit (QIAGEN, Tokyo, Japan), and BioRobot EZ2 (QIAGEN). Total RNA (1 μg) was subjected to reverse transcription using Omniscript Reverse Transcriptase (QIAGEN). PCR analysis was performed using TaKaRa Ex Taq Hot Start Version (Takara, Kyoto, Japan), with the following primers purchased from Sigma-Aldrich Japan: *SEAP* [GenBank accession no. U89937; National Center for Biotechnology Information (NCBI; http://www.ncbi.nlm.nih.gov/Genbank/index.html)]: forward primer 5′-CAGGACATCGCTACGCAGCTCATCT-3′, reverse primer 5′-GTAAGCCTGCTTT CATGATGACCA-3′; *SEAP* (for nested PCR): forward primer 5′-AACATGGACATTGACGTGATCCTAG-3′, reverse primer 5′-TCTCGTATTTCATGTCTCCAGG CTC-3′; *Cyp1a1* [Gene ID: 13076 (NCBI; http://www.ncbi.nlm.nih.gov/sites/entrez?db=gene&cmd=search&term=)]: forward primer 5′-CAGATGATAAGGTCATCACGA-3′, reverse primer 5′-TTGGGGATATAGAAGCCATTC-3′; *Gapdh* (Gene ID: 14433; NCBI): forward primer 5′-ACCACAGTCCATGCCATCAC-3′, reverse primer 5′-TCCACCACCCTGTTGCTGTA-3′.

### Statistical analysis

Data are expressed as mean ± SE (*n* ≥ 4). Statistical analysis was performed using the nonparametric Mann–Whitney *U*-test to compare data among the different groups. A *p*-value < 0.05 was considered statistically significant.

## Results

### Generation of transgenic sensor mice

**Figure 1:**
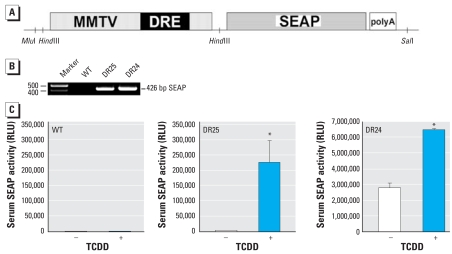
Generation of DRE-based sensing via secreted alkaline phosphatase (DRESSA) mice. Abbreviations: DRE4, a fragment from the mouse *Cyp1a1* gene promoter that contains four DREs; MMTV, a fragment from the mouse mammary tumor virus promoter; RLU, relative light unit; WT, wild-type mice. (*A*) Schematic representation of the reporter construct introduced into mice. (*B*) Integration of the *SEAP* gene in DRESSA25 (DR25) and DRESSA24 (DR24) mice examined using PCR analysis. (*C*) Responses of serum SEAP activity before (−) or 3 days after (+) oral exposure to 5 μg/kg bw TCDD. Numbers of mice tested: WT (*n* = 4), DR25 (*n* = 5), and DR24 (*n* = 4). Data are presented as mean ± SE. *Statistically significant difference, *p* < 0.05.

We previously reported a fast, sensitive bioassay, DRESSA, that can detect and quantify the levels of halogenated and polycyclic aromatic hydrocarbons (Kasai et al. 2004, 2005). In this approach, a murine hepatoma cell line was stably transfected with a *SEAP* gene under the control of DRE, thereby creating reporter cells. Using the same gene construct (Figure 1A), we generated transgenic sensor mice DRESSA that can produce SEAP under the control of AhR. Briefly, the regulatory sensor sequence consists of two parts: *a*) a 484-bp fragment from the upstream region of the mouse *Cyp1a1* gene that contains four DREs, and *b*) a 1,330-bp fragment from part of the mouse mammary tumor virus promoter lacking glucocorticoid-responsive enhancers. The latter fragment alone does not have the transacting potential but substantially enhances activation of DRE by AhR–ligand complexes (Kasai et al. 2006b). Two transgenic lines, DRESSA24 and DRESSA25, were established, and integration of the transgene was confirmed by PCR analysis (Figure 1B). To examine *in vivo* responses of the sensor mice to AhR agonists, we administered 5 μg/kg bw TCDD orally to wild-type and DRESSA mice. After 3 days, blood was sampled from the tail veins, and serum levels of SEAP were evaluated. As shown in Figure 1C, wild-type mice exhibited background levels of serum SEAP activity [973 ± 232 relative light unit (RLU), mean ± SE], and it was not increased by TCDD (1,193 ± 307 RLU; Figure 1C). DRESSA25 exhibited a low level of basal SEAP activity (4,297 ± 199 RLU), which was markedly elevated (approximately 50-fold) in response to TCDD (226,260 ± 69,333 RLU; Figure 1C). In contrast, DRESSA24 mice showed high levels of basal SEAP activity (Figure 1C). Constitutive expression of *SEAP* mRNA was observed in all organs tested, including brain, lung, heart, liver, spleen, kidney, muscle, and adipose tissue [Supplemental Material, Figure 1 (online at http://www.ehponline.org/members/2007/10722/suppl.pdf)], which may be caused by integration of the transgene downstream of some housekeeping gene promoter(s). However, responsiveness to TCDD was still preserved in DRESSA24; that is, after the administration of TCDD, serum levels of SEAP increased from 2,786,626 ± 309,783 RLU to 6,577,877 ± 31,885 RLU (Figure 1C).

### Responses of DRESSA mice to AhR agonists

**Figure 2:**
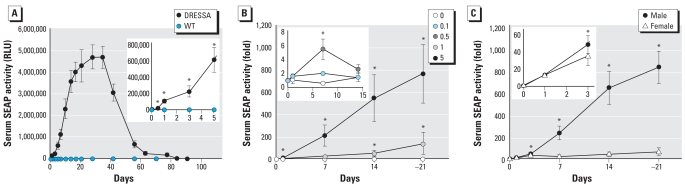
Kinetics of serum SEAP in DRESSA mice exposed to TCDD. (*A*) Serum SEAP activity in wild-type (WT, *n* = 2) and DRESSA25 (DRESSA, *n* = 5) mice after oral administration of 5 μg/kg bw TCDD. (*B*) Dose-dependent responses of serum SEAP in DRESSA25 mice exposed to 0.1–5 μg/kg bw TCDD. Fold induction of SEAP vs. basal level is shown (*n* = 4–5 mice). (*C*) Sex-dependent difference in the response to TCDD. Age-matched male (*n* = 27) and female (*n* = 22) DRESSA25 mice were exposed to 5 μg/kg bw TCDD, and activity of serum SEAP was evaluated. Data are presented as mean ± SE. *Statistically significant difference, *p* < 0.05.

We examined kinetics of serum SEAP in male DRESSA25 mice after oral administration of 5 μg/kg bw TCDD. As shown in Figure 2A (inset), significant elevation of serum SEAP activity was observed within 12 hr of administration of TCDD (4.3 ± 0.6-fold; *p* < 0.05). Unexpectedly, during the course of the study, the level of SEAP continuously increased until day 28 without additional exposure to TCDD. The induction rate at day 28 was approximately 1,100-fold over the basal level. The level of SEAP then gradually declined, but significant elevation of SEAP was still detectable until day 84. Administration of corn oil alone did not cause elevation of serum SEAP (data not shown). Throughout the experiments, wild-type mice administered TCDD did not show alterations in the level of serum SEAP (Figure 2A).

We examined dose-dependent responses of DRESSA mice to TCDD. DRESSA25 mice were orally administered 0.1–5 μg/kg bw TCDD, and we evaluated serum SEAP activity until day 21. As with 5 μg/kg bw TCDD, administration of 1 μg/kg bw TCDD also caused significant elevation of serum SEAP at days 14 and 21 (Figure 2B). The induction rate at day 21 was approximately 140-fold over that of basal level. In contrast, administration of 0.5 μg/kg bw TCDD caused only modest, transient increases in SEAP at day 7 (5.4 ± 1.4-fold; *p* < 0.05), and the level returned to basal level at day 14. The 0.1-μg/kg bw group showed no significant responses to TCDD (Figure 2B, inset). These results suggest that in DRESSA mice, the level of serum SEAP increases dose dependently in response to TCDD and that the detection limit of TCDD is approximately 0.5 μg/kg bw.

Adult male DRESSA mice were used in the experiments described above. Previous reports, however, indicate that sex-dependent differences in response to xenobiotics may be present in guinea pigs, macaques, and mice (Enan et al. 1996; Jones et al. 1991). We therefore compared responses of male and female DRESSA25 mice administered 5 μg/kg bw TCDD. Interestingly, there was no difference in the increase in serum SEAP between males and females at day 1, that is, an approximate 13-fold elevation in both groups (Figure 2C, inset). However, although significant elevations of serum SEAP continued in females for at least 3 weeks, the degree of elevation was lower than that in males (Figure 2C). Induction at day 21 was 845 ± 149-fold in males and 67 ± 34-fold in females. These results suggest that male mice respond more sensitively to aromatic hydrocarbons and are suitable as sensing animals to detect environment pollution.

**Figure 3:**
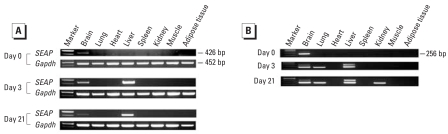
RT-PCR analysis of *SEAP* expression in various organs after oral administration of TCDD. DRESSA25 mice were administered 5 μg/kg bw TCDD. Before and after 3 and 21 days, various organs were subjected to RT-PCR (*A*) and nested PCR (*B*) analyses of *SEAP*. Glyceraldehyde 3-phosphate dehydrogenase (*Gapdh*) was used as an internal control. Experiments were repeated 3–4 times with similar results.

To identify major organs responsible for the production of SEAP, we exposed DRESSA25 mice to 5 μg/kg bw TCDD, po, and after 3 and 21 days, several organs—brain, lung, heart, liver, spleen, kidney, muscle, and adipose tissue—were subjected to RT-PCR analysis of *SEAP*. Before exposure to TCDD, a low level of *SEAP* mRNA was detectable in the brain but not in other organs tested (Figure 3A, day 0). At days 3 and 21, expression of *SEAP* was induced markedly in the liver and modestly in the brain (Figure 3A). In other organs, induction of *SEAP* mRNA was undetectable. We further tested expression of *SEAP* using nested RT-PCR analysis and found that low levels of *SEAP* were also induced in the lung and kidney (Figure 3B).

**Figure 4:**
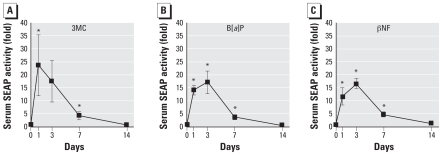
Responses of DRESSA mice to polycyclic aromatic hydrocarbons. DRESSA25 mice were orally administered 10 mg/kg bw 3MC (*n* = 5), B[*a*]P (*n* = 4), or βNF (*n* = 4). Levels of serum SEAP were evaluated on days 1, 3, 7, and 14. Data are presented as mean ± SE. *Statistically significant difference, *p* < 0.05.

AhR is activated not only by halogenated aromatic hydrocarbons (for example, dioxins) but also by polycyclic aromatic hydrocarbons. We tested responses of DRESSA mice to other AhR ligands, especially polycyclic aromatic hydrocarbons. We orally exposed DRESSA25 mice to 10 mg/kg bw 3MC, B[*a*]P, or βNF and evaluated levels of serum SEAP. As shown in Figure 4, significant elevation of SEAP (10- to 20-fold) was observed in all groups with a peak at days 1–3. The levels of serum SEAP declined thereafter, and significant elevation was not observed at day 14.

### Monitoring of air pollution caused by cigarette smoke

**Figure 5:**
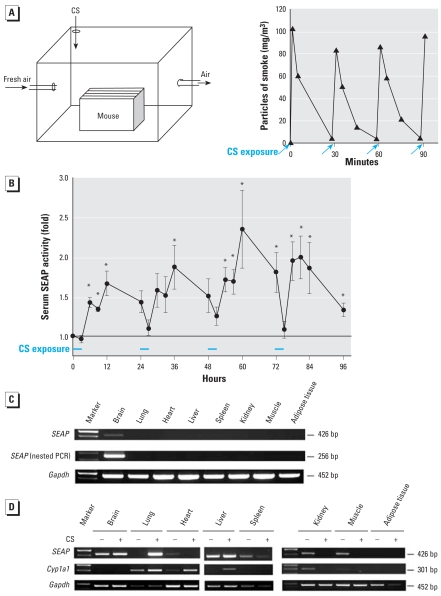
Monitoring of air pollution caused by cigarette smoke using DRESSA mice. CS, cigarette smoke. (*A*) Schematic representation of the experimental smoking room (left). DRESSA mice were placed in the room continuously ventilated with fresh air. Mainstream smoke (250 mL) was injected into the room every 30 min for a total of 6 times/day, and kinetics of air pollution (density of smoke particle) was evaluated successively (right). (*B*) Kinetics of serum SEAP in DRESSA mice during recurrent exposures to polluted air. DRESSA25 mice (*n* = 4) were placed in the smoking room and exposed to the air polluted by cigarette smoke for 3 hr. After exposure, mice were maintained for the following 21 hr outside the smoking room. This procedure was repeated everyday for up to 4 days. Activity of serum SEAP was evaluated 0, 3, 6, 9, and 21 hr after exposure to polluted air. Data are presented as mean ± SE. (*C*) Expression of *SEAP* mRNA in various organs of DRESSA25 mice after exposure to polluted air. DRESSA25 mice were exposed to smoke-polluted air for 3 hr and left outside the room for 1 hr. Indicated organs were subjected to RT-PCR and nested PCR analyses of *SEAP*. (*D*) Expression of *SEAP* and *Cyp1a1* in various organs after exposure to polluted air in DRESSA24 mice. DRESSA24 mice were exposed to smoke-polluted air (+) or fresh air (–) and various organs were subjected to RT-PCR analysis of *SEAP* and *Cyp1a1.* *Statistically significant difference, *p* < 0.05.

Using the *in vitro* DRESSA bioassay (Kasai et al. 2004, 2005), we recently reported that cigarette smoke contains high levels of AhR agonists and strongly activates the AhR–DRE pathway (Kasai et al. 2006a). Because cigarette smoke contains halogenated and polycyclic aromatic hydrocarbons (Lofroth and Zebuhr 1992; Muto and Takizawa 1989), we examined whether air pollution caused by cigarette smoke can be detected and monitored directly and successively using the established mice. For this purpose, DRESSA25 mice were placed in an experimental smoking room that was ventilated continuously by fresh air (Figure 5A). Mainstream smoke was injected into the room every 30 min for a total of 6 times/3 hr. Kinetics of the smoke particle density measured by a digital aerosol monitor is shown in Figure 5A. Chemiluminescent assay revealed that after the exposure to the polluted air, serum levels of SEAP were significantly elevated. The level peaked at 12 hr (9 hr after the last exposure) and returned to basal level after 24 hr. This result was reproducible, and after each exposure to the polluted air, DRESSA25 mice consistently exhibited similar kinetics of serum SEAP (Figure 5B). In contrast, DRESSA25 mice placed in the room for 3 hr without exposure to polluted air showed no increases in the level of serum SEAP [Supplemental Material, Figure 2 (online at http://www.ehponline.org/members/2007/10722/suppl.pdf)].

To identify organs responsible for the sensing of air pollution, we sacrificed DRESSA25 mice 1 hr after the 3-hr exposure and subjected various organs to RT-PCR analysis of *SEAP*. However, we could not detect *SEAP* mRNA in the organs tested except for the brain, which has some basal expression (Figure 5C). We therefore tried nested RT-PCR analysis, but the result was the same (Figure 5C) because the induction of *SEAP* in response to air pollution was much lower than that induced by TCDD. To overcome this problem, we used another transgenic line, DRESSA24. The advantage of using this line is that it possesses not only responsiveness to AhR agonists but also substantial basal expression of *SEAP* in various organs [Supplemental Material, Figure 1 (online at http://www.ehponline.org/members/2007/10722/suppl.pdf)]. Compared with wild-type mice, increased activity of SEAP was also detectable in all organs, as shown in Supplemental Material, Figure 3 (online at http://www.ehponline.org/members/2007/10722/suppl.pdf). Using the DRESSA24 mice exposed to polluted air in the smoking room, we found that induction of *SEAP* was observed predominantly in the lung but not in other organs tested (Figure 5D). This was in contrast to mice orally exposed to TCDD, which induced *SEAP* predominantly in the liver (Figure 3A). Induction of *SEAP* in the lung correlated with induction of *Cyp1a1*, an AhR-dependent gene (Figure 5D). Notably, induction of *Cyp1a1* was also observed in some of the other organs tested. Figure 5D shows that expression of *SEAP* and *Cyp1a1* in some of the organs decreased after exposure to polluted air, but the result was not consistent with other experiments using distinct pairs of mice (data not shown). These results suggest that the lung is the major sensing organ responsible for production of SEAP in response to air pollution.

## Discussion

Several groups previously reported establishment of transgenic animals responsive to aromatic hydrocarbons. For example, Mattingly et al. (2001) generated transgenic zebrafish that express green fluorescent protein (GFP) under the control of the human *CYP1A1* promoter/enhancer. To enable total, accurate risk assessment of environmental pollution in humans, however, use of land mammals is inevitable. Some investigators reported dioxin-responsive transgenic mice using chloramphenicol acetyl-transferase (CAT), β-galactosidase, GFP, or luciferase as a reporter protein (Galijatovic et al. 2004; Jones et al. 1991; Operana et al. 2007; Willey et al. 1998). However, these mice have been used only to investigate activation of AhR or the *Cyp1a1* promoter in specific organs or tissues after oral or ip administration of halogenated and polycyclic aromatic hydrocarbons. Although the first transgenic mice were generated more than 15 years ago, there are no reports of successful monitoring of environmental pollution, especially air pollution, using transgenic mice, conceivably because of the following reasons. First, in transgenic mice, induction of reporter proteins by aromatic hydrocarbons is weak and only up to severalfold above basal levels, even if the animals were treated with high levels of toxicants (Galijatovic et al. 2004; Operana et al. 2007; Willey et al. 1998). Second, to evaluate quantitatively the level of β-galactosidase, GFP, or luciferase, reporter mice must be sacrificed and/or internal organs exposed or excised for analyses. This approach does not allow for continuous, real-time monitoring of environmental pollution using identical animals. *In vivo* bioluminescence imaging of luciferase activity may be useful for overcoming this problem (Honigman et al. 2001), but in this case, a particular imaging apparatus, general anesthesia of animals, and administration of luciferin are required for assessment. Third, CAT, β-galactosidase, and GFP are stable proteins, and their half-lives are 20–50 hr, which is not suitable for sensitive, real-time assessment of pollutant levels. In contrast, the SEAP-based system has several advantages. First, only 5 μL serum is sufficient for measurement of SEAP activity. Sacrifice of animals or sampling of organs is not required, an advantage for animal welfare and cost performance. Second, activity of SEAP can be measured quickly, sensitively, and quantitatively with conventional chemiluminescent systems (Kasai et al. 2005, 2004). The cost required is much lower than that of other methods, including luciferase-based systems. Third, *in vivo* half-life of serum SEAP in mice is approximately 2 hr (Hiramatsu et al. 2007), which allows for real-time assessment of the level of pollutants, as shown in the present article. Although secreted luciferase may also have a short half-life and can be detected sensitively by conventional chemiluminescent methods, it has an obvious limitation for *in vivo* use. That is, activity of luciferase cannot be mesured in the presence of serum albumin and cannot serve as an *in vivo* reporter protein, as we reported previously (Hiramatsu et al. 2005).

In the present article we provide evidence that single, oral exposure to 5 μg/kg bw TCDD caused dramatic, long-term activation of the AhR–DRE pathway. A previous study showed that oral administration of 1–10 μg/kg bw TCDD caused accumulation mainly in the liver and adipose tissue in mice (Diliberto et al. 1995). It is, in part, consistent with our result that activation of the AhR–DRE pathway was observed mainly in the liver in TCDD-exposed DRESSA mice. However, Diliberto et al. (1995) also showed that the level of TCDD in the liver declined in a time-dependent manner. In contrast, we found that whole-body activity of the AhR–DRE pathway, indicated by serum SEAP, increased progressively up to days 28–35. Currently, the reason for the discrepancy is unclear. The fact that the whole-body half-life of TCDD is less than the 28–35 days when SEAP reaches a maximum response indicates a possibility that the whole-body level of TCDD may not correlate with the whole-body response to TCDD. In this study, we demonstrate that the sensing potential of male mice was higher than that of female mice. This is consistent with a previous report that hepatic activation of the *Cyp1a1* promoter by 3MC was approximately 10-fold greater in males than in females in *Cyp1a1*–CAT transgenic mice (Jones et al. 1991). Although, generally, there are not marked sex differences in the toxic potency of dioxin, previous studies showed that susceptibility to dioxin was higher in males than in females in some laboratory animals. For example, both male and female guinea pigs exhibit the same physical and biochemical responses to TCDD, but its toxicity in females is evident only after longer exposures to higher dosages of the chemical (Enan et al. 1996). The sex-dependent difference may be ascribed to influences of sex steroids. A previous study showed that in murine hepatoma cells, estradiol suppressed the AhR–DRE pathway triggered by dioxin through AhR but not through the ER (Jeong and Lee 1998).

Environmental pollution by aromatic hydrocarbons is monitored currently by physicochemical evaluation of individual compounds. However, this conventional approach is not adequate for real risk assessment of the toxicologic influences of aromatic hydrocarbons on humans. First, although environmental pollutants enter the body via multiple routes, current assessment does not consider exposure routes; that is, route-dependent differences in absorption and metabolism of pollutants. Notably, the magnitude of responses to aromatic hydrocarbons depends largely on the route of exposure (Operana et al. 2007). Second, the physicochemical evaluation is also unable to consider synergism and antagonism among various chemicals in environments. The most typical example is cigarette smoke, which contains more than 4,800 chemicals. Cigarette smoke contains a number of halogenated and polycyclic aromatic hydrocarbons such as polychlorinated dibenzo-*p*-dioxins, polychlorinated dibenzofurans, coplanar polychlorinated biphenyls, and B[*a*]P (Lofroth and Zebuhr 1992; Muto and Takizawa 1989). However, previous assessment with gas chromatography–mass spectrometry showed that the levels of individual chemicals in cigarette smoke were very low and within permissible ranges (Aoyama et al. 2003). In contrast, using the *in vitro* DRESSA bioassay, we demonstrated that cigarette smoke has high levels of the dioxin-like potential for triggering the AhR–DRE pathway (Kasai et al. 2006a). Thus, the current method for monitoring environmental pollutants has a crucial limitation; that is, the method cannot assess accumulative, integrative, and accurate risk in humans exposed daily to various environmental pollutants through multiple routes. To overcome this problem, the transgenic sensor mice described here would open a new window toward direct, realistic monitoring of environmental contamination from dioxin-like compounds.

However, it must be noted that the detection sensitivity of the established mice is not extremely high; that is, the level of serum SEAP was not altered in response to 0.1 μg/kg bw TCDD that might be toxic to mammals. Furthermore, with this monitoring system, activity of serum SEAP could be affected by ingestion of some AhR agonists and/or antagonists in foods (Amakura et al. 2003a, 2003b), as well as by alterations in the levels of putative, endogenous AhR ligands, including tryptophane photooxidation products, lypoxin A4, indirubin, bilirubin, biliverdin, and an indole derivative (Puga et al. 2005). Influences of these exogenous and endogenous AhR ligands should be considered carefully, when the DRESSA mice are used for monitoring of environmental pollution.
